# Development of the Ultralight Hybrid Pneumatic Artificial Muscle: Modelling and optimization

**DOI:** 10.1371/journal.pone.0250325

**Published:** 2021-04-22

**Authors:** Seonggun Joe, Massimo Totaro, Hongbo Wang, Lucia Beccai

**Affiliations:** 1 Soft BioRobotics Perception, Istituto Italiano di Tecnologia (IIT), Genova, Italy; 2 The BioRobotics Institute, Scuola Superiore Sant’Anna, Pontedera, Italy; Boston University, UNITED STATES

## Abstract

Pneumatic artificial muscles (PAMs) are one of the key technologies in soft robotics, and they enable actuation in mobile robots, in wearable devices and exoskeletons for assistive and rehabilitative purposes. While they recently showed relevant improvements, they still present quite low payload, limited bandwidth, and lack of repeatability, controllability and robustness. Vacuum-based actuation has been recently demonstrated as a very promising solution, and many challenges are still open, like generating at the same time a large contraction ratio, and a high blocking force with enhanced axial stiffness. In this paper, a novel Ultralight Hybrid PAM (UH-PAM), based on bellow-type elastomeric skin and vacuum actuation, is presented. In particular, open-cell foam is exploited as a structural backbone, together with plastic rings, all embedded in a thin skin. The design and optimization combine numerical, analytical, and experimental data. Both static and dynamic analysis are performed. The weight of the optimized actuator is only 20 g. Nevertheless, a contraction ratio up to 50% and a maximum payload of 3 kg can be achieved. From a dynamic point of view, a rise time of 0.5 s for the contraction phase is observed. Although hysteresis is significant when using the whole contraction span, it can be reduced (down to 11.5%) by tuning both the vacuum range and the operating frequency for cyclic movements. Finally, to demonstrate the potentiality of this soft actuation approach, a 3 DoFs Stewart platform is built. The feasibility of performing smooth movements by exploiting open-loop control is shown through simple and more complex handwriting figures projected on the XY plane.

## 1. Introduction

With the rapid developments in soft robotics [1,2], various pneumatic artificial muscles (PAMs) have been developed for a wide range of applications, like wearable and rehabilitation devices [3,4], and medical equipment [5]. Even though pneumatic actuation requires bulky air compressors, it has some promising characteristics compared to other technologies (*i*.*e*., electric motor or tendon driven systems [6], piezoelectric actuators [7], dielectric elastomers [8], shape memory alloys [9], polymers or ionic polymers [10,11]). In general, PAMs feature simplicity, large contraction ratio, robustness, high power to mass ratio [12], and easy fabrication. With the recent remarkable advances in additive manufacturing, the molds for casting soft materials can be conveniently created, even if having complex 3D geometry [13]. Moreover, direct 3D printing of PAMs (similarly to other soft actuators) is becoming a feasible option, when carefully tuning the printing parameters for soft materials [14]. Indeed, such 3D printing technology enables programmable multidimensional actuations that feature complex architectures and tunable stiffness by using multi-materials [15–17]. Nevertheless, main challenges are still to be met, such as large pulling force, deformation, and fast response [18,19].

One of the most popular PAM is the McKibben muscle (invented in 1950s), which usually consists of an elastic bladder covered by inextensible fiber braids/meshes to constrain and guide the deformation of the bladder itself [20,21]. The McKibben actuator can generate a linear contraction with large payloads, and can achieve high power efficiency. For instance, Caldwell *et*. *al*. presented a McKibben PAM design that achieved a very high power to weight ratio, *i*.*e*., 1.5 kW/kg at 200 kPa, and 3 kW/kg at 400 kPa [22]. Recently, Skorina *et*. *al*. presented reverse PAMs (rPAMs), made of silicone rubber radially constrained by symmetrical double-helix threading, operating similarly to PAMs but with a reverse actuation direction [23]. The rPAM can be precisely controlled without bulky flow control valves and extensive computer hardware, and its behavior can be predicted by means of an analytical model. Indeed, a quasi-static analytical model was built, using both the constraint model for the helical threads and the Ogden material model for silicone material properties. As a result, the force that rPAM can produce at a given length was obtained by a resultant force between the helical constraint force and the force due to internal material stresses. The experimental and theoretical results showed a non-linear behavior, while they matched very well at low-pressure range (0 to 70 kPa) when no weight was imposed. However, this reinforced structure still requires a high pressure (up to 200 kPa) to satisfy the desired displacement or force. Indeed, the large actuating force, with large contraction or elongation ratio, strongly depends on the mechanical behavior of the embodied materials.

On the other hand, the so-called Peano actuator, consisting of a set of tubes arranged side-by-side, can implement not only linear contraction and torsional motion, by using very low pressure (10 kPa), but also high blocking force (25 N) at low pressures (30 kPa) [24]. However, the contraction ratio is limited (36%), because of the cylindrical geometry of the inflated membranes. The Peano-HASEL actuator has a similar cylindrical structure, and it combines the strengths of fluidic and electrostatic actuators. It can be linearly contracted without rigid frames, pre-stretching, or stacked configuration, but it requires high voltage (12 kV), and it leads to quite a small payload (500 g) [25].

To overcome the limited contraction ratio, Fluid-driven Origami-inspired Artificial Muscles (FOAMs) were proposed [26,27]. FOAMs feature powerful actuation stress, large deformation, and low-cost fabrication. Indeed, they can be programmed for multidimensional actuation, by means of folding lines built in thin layered structures with zigzag geometry. This approach enables an extremely large contraction ratio (> 90%). At the same time, a fast fabrication process (within 10min) having a very low cost (less than 1 USD) can be used [26]. Due to such characteristics, FOAMs can produce a very high-power density (up to 2 kW/kg). In recent work, it was shown how a particular kind of FOAMs, *i*.*e*., Origami-based Vacuum Pneumatic Artificial Muscle (OV-PAM), can generate a large payload (up to 400 N) with a dramatically improved power density (up to 300 kW/kg) [27]. However, the repeated actuation can cause material failures, lack of mechanical coupling between the different layers, or stress concentration along the folds and vertices [28]. An optimization of structure design (*e*.*g*. specific hole patterning [28–30]) was proposed to release stress concentration at the cost of having more fragile structures (30).

Recently, various Vacuum-Actuated Muscle-inspired Pneumatic structures (VAMPs) have been developed by using elastomeric materials (*e*.*g*. silicone rubber, flexible resin). Yang *et*. *al*. presented actuators that can generate linear contraction and torsional motion due to the buckling of their structural elastomeric beams [31]. Similarly, Vacuum-powered Soft Pneumatic Actuators (V-SPAs) with a foam core were developed for building 3 degrees-of-freedom (DoFs) joints. Multiple V-SPA modules were connected in series, and demonstrated in a multifunctional soft robot with a response time in the order of few seconds [32]. Recently, Jiao *et*. *al*. [33] expanded this approach and developed the Vacuum-Powered Soft Pneumatic Twisting Actuator (V-SPTA) that has two DoFs. Various demonstrations were introduced by exploiting V-SPTA promising characteristics, *i*.*e*., diverse functionality, high load capacity, minimized design complexity, and low cost. In general, systems using negative pressure show greater safety, compactness, robustness and fast response compared with PAMs actuated by a positive pressure. However, in case of soft elastomeric structures, both the vacuum itself and the buckling strength can easily limit the maximum contraction and the blocking force. Indeed, the analysis of the constituent materials and of the mechanical behavior of full elastomeric structures are essential to optimize the PAMs design.

Among all possible designs, bellow-type structures can generate a large contraction ratio, comparable to mechanical compression springs. Tawk *et*. *al*. [34] presented a Linear SOft Vacuum Actuator (LSOVA) fabricated by 3D printing. In particular, connecting in series five chambers (5C-LSOVA) the contraction ratio reached 51.5% with 27.66 N of blocking force. However, a large hysteresis of 70% was observed, because of the buckling of the thin walls. Similarly, Digumarti *et*. *al*. presented a Hyper-Elastic Bellow (HEB) using both positive and negative pressure. In this case, the deformation reached 450% and 80% in longitudinal and radial direction, respectively [35]. Moreover, by adding rigid parts (rings) periodically spaced along its length, Felt *et*. *al*. [36] showed that a bellow type soft PAM, made of a tubular thin membrane, can lift a high payload of 21.35 N at -5.52 kPa. Such investigation on V-SPAs has demonstrated that this kind of actuation can enable high contraction ratio and blocking force capability, opening the way to new PAM concepts. Indeed, many other important features should be still addressed, like the axial stiffness, which was not analyzed for this class of PAMs.

In this work, we present an innovative Ultralight Hybrid PAM (UH-PAM) that exploits open-cell foam and thin rigid rings as a structural backbone, all embedded in a thin elastomeric skin. The design is conceived to generate a large contraction ratio, and a high blocking force with enhanced axial stiffness. Firstly, the mechanical behavior of a bellow-type elastomeric skin under vacuum is investigated (building on a preliminary study [37]), and the optimization of its structure is performed through Finite Element Method (FEM) simulations. Then, different PAM structures are built, mainly by casting using 3D printed molds, and both static and dynamic characterizations are addressed, in order to optimize the UH-PAM configuration. Finally, different platforms integrating the UH-PAM are implemented. In particular, a 3 DoFs Stewart platform is developed by exploiting open-loop control and simplified geometry mapping algorithms. In this way, we show the potential of this approach, as well as providing more insights for future robotic developments.

## 2. Materials and methods

### 2.1 Concept design of the bellow-type PAM

**Fig 1:**
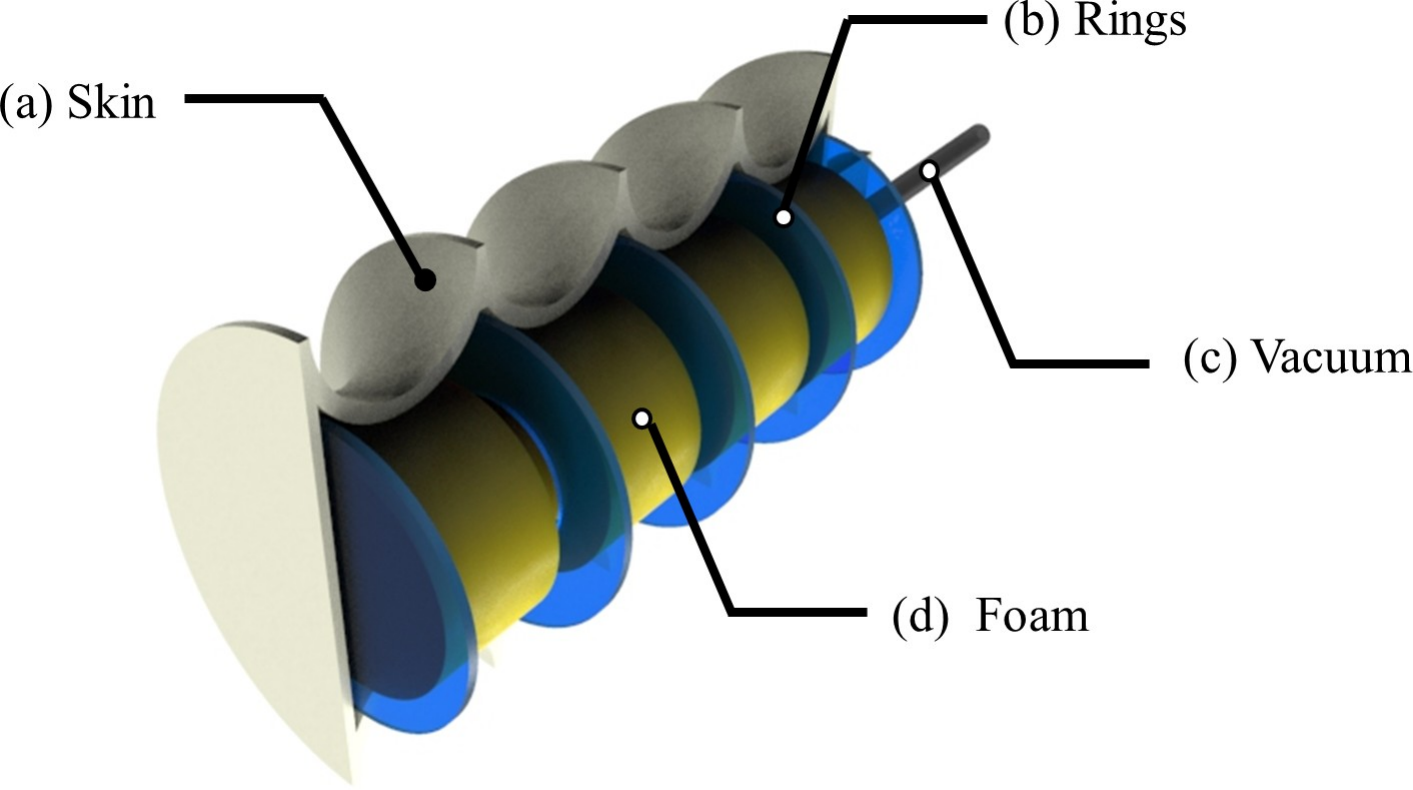
Concept design of the proposed pneumatic artificial muscle: (a) bellow-type skin; (b) rigid rings; (c) vacuum line; (d) open-cell foam cylinders.

As shown in Fig 1, the proposed vacuum-based PAM consists of a hyperelastic skin that encases open-cell foam cylindrical segments and plastic rings. As it will be detailed in the following sections, the bellow configuration allows the PAM to achieve high contraction ratio and blocking force, and the open-cell foam provides mechanical support to external load and/or weight, which also guarantees structure stability during actuation. In addition, the plastic rings prevent undesired structural deformation (*i*.*e*., buckling) by strengthening the stiffness along the longitudinal axis (or axial stiffness).

### 2.2 Design and optimization

Our objective in designing the soft actuator was to maximize the contraction ratio while maintaining a proper axial stiffness, by optimizing its structure. The bellow axial stiffness is function of its dimensions and material composition (*i*.*e*., diameter, thickness, number of convolutions, pitch, height, method of reinforcement, etc.). The axial spring rate (proportional to axial stiffness) is an important parameter to be considered in PAM buckling analysis, such to determine the structural stability upon external force.

The design process consisted in different phases. At first, different designs were investigated by means of FEM simulations, by considering only the external elastomeric skin with a bellow structure, in order to evaluate how each PAM was deformed in the longitudinal direction by a negative pressure. Then, by exploiting the optimal geometry, different actuators were fabricated to investigate some possible strategies for further axial stiffness reinforcement.

**Fig 2:**
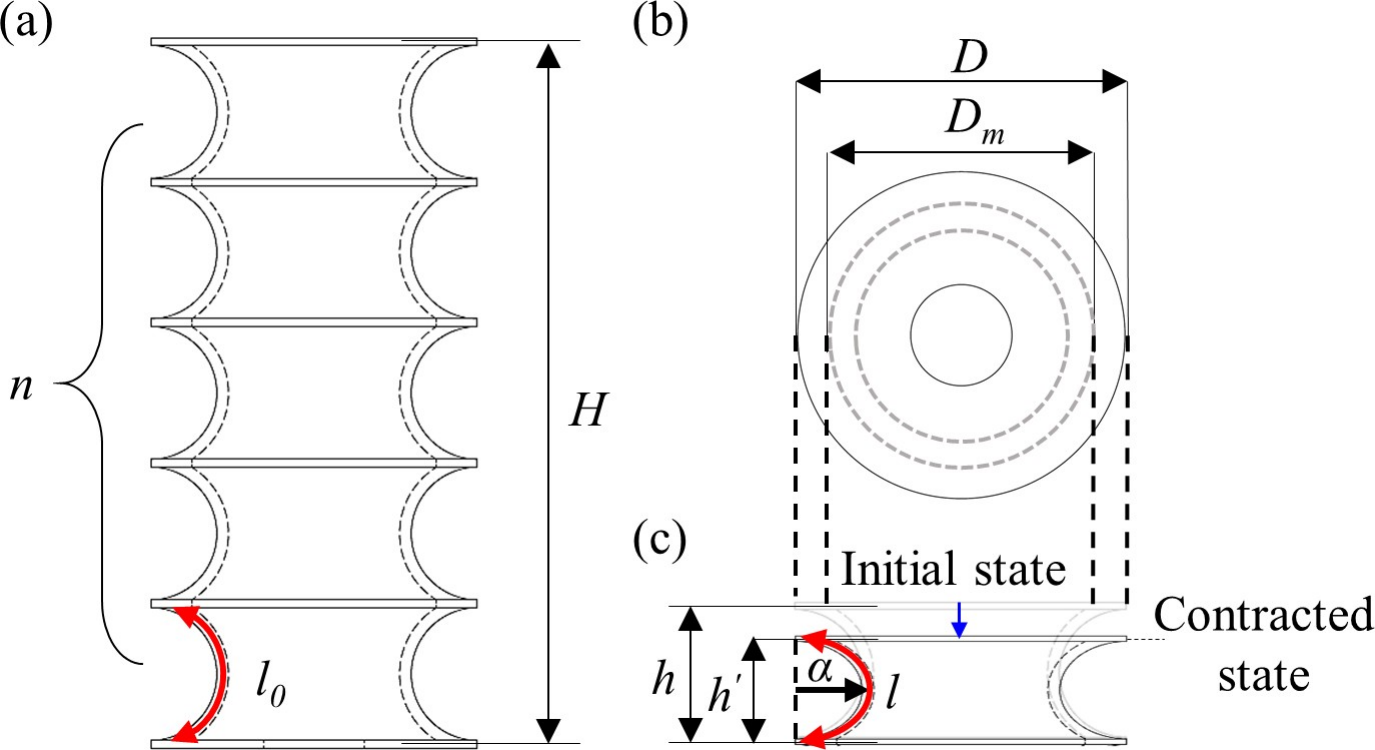
Design parameters: (a) a bellow structure at the initial state with null applied vacuum; (b) top view of bellow structure; (c) a single segment in the contracted state induced by vacuum.

The different bellow-type structures were designed by considering that the convoluted length (or number of convolutions) is the parameter that dominantly affects the buckling failure [38]. For this reason, all other design parameters (*i*.*e*., the total length, the mean and the outer diameter and thickness of bellow) were kept constant. Different bellow convolutions [*n*] were inserted in the structure, and consequently the convoluted lengths [*h*] were defined. Table 1 summarizes all the parameters used in the design (shown in Fig 2) of all considered skin structures (cylinder and bellows) starting from a cylinder with diameter of 34 mm and length of 78 mm.

**Table 1:** Nomenclature of the bellow skin.

Parameter	Description
*D* [mm]	Diameter
*D*_*m*_ [mm]	Mean diameter
*n* [turns]	Number of convolutions
*l*_*0*_ [mm]	Arc length at initial state
*l* [mm]	Arc length at contracted state
*H* [mm]	Total length
*h* [mm]	Length of single segment at initial state
*h*^*’*^[mm]	Length of single segment at contracted state
*α* [mm]	Length of major axis

In addition to open-cell foam and thin plastic rings, two different elastomers for the skin (Dragon Skin^TM^ 10 and 30, Smooth-On Inc., Macungie, PA, USA) were considered. Each prototype was analyzed by compression tests, and the PAM structure having the largest contraction ratio, buckling pressure and blocking force, was identified.

#### 2.2.1 FEM modeling of the skin

A FE model was created in ANSYS 19.2 (ANSYS Inc., Canonsburg, PA, USA) for static structural analysis, in order to evaluate the deformation and strain energy of the skin under pressure loading. The material properties of the skin were defined by the incompressible Mooney-Rivlin hyperelastic model [39] using nine parameters (listed in S1 Table) that were obtained through curve fitting of uniaxial elongation data of Dragon Skin^TM^ 10.

**Fig 3:**
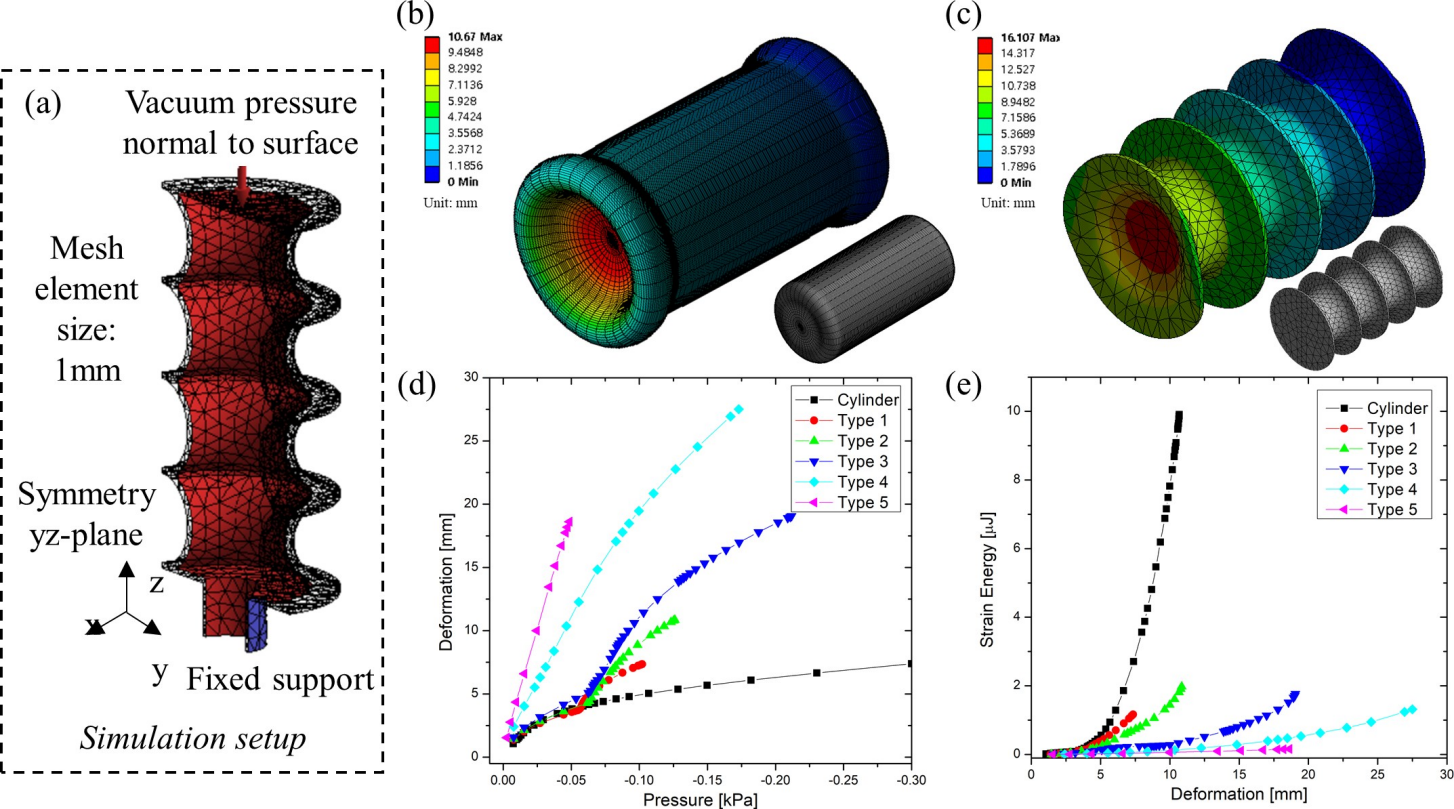
FEM results: a) simulation setup of half structure, where planar symmetry was exploited; (b) deformation of a simple cylindrical skin structure with -0.62 kPa of applied vacuum; c) deformation of bellow Type 3 with -0.21 kPa of applied vacuum; (d) pressure versus deformation graphs for all bellow types; (e) deformation versus strain energy graphs for all bellow types. In (b) and (c): the color legends show deformation values in mm; the gray models show the relative meshes used for the simulations.

To reduce computation time, only half of the structure was simulated in an axis symmetry model (as shown in Fig 3(A)). In addition, the progressive deformations of the structure were calculated in multiple steps, allowing the software to add sub-steps in an iterative fashion when the convergence failed. The default mesh element size was set to 1 mm. Each bellow-type was defined by the number of convolutions with respect to the convoluted length. Type 1 to Type 5 had different convolution numbers [*n*] of 2, 3, 4, 8, and 14, respectively, with the convoluted lengths varying from 4.14 mm to 35 mm. As an example of the obtained results, the deformation of a simple cylindrical structure and Type 3 bellow are shown in Fig 3B and 3C, respectively. In addition, the graphs of Fig 3D and 3E show the deformation and the strain energy as a function of the negative pressure, respectively, while Table 2 summarizes the values of key parameters for all simulated structures.

**Table 2:** Simulation results of the different types of skin.

	Cylinder	Type 1	Type 2	Type 3	Type 4	Type 5
*n*	0	2	3	4	8	14
*h’* [mm]	-	35	23	17	8	4.14
Strain Energy [μJ]	9.908	1.152	1.198	1.77	1.32	0.16
Stress [kPa]	46.89	2.286	2.263	5.82	2.87	1.60
Max. deformation [mm]	10.69	7.349	10.85	19.058	27.505	18.621

Each skin type showed different deformations at the same vacuum level. For instance, Type 5 was deformed up to 18.6 mm at -0.05 kPa, while the deformation of the cylinder was only 3.8 mm at the same vacuum pressure. The strain energy of Type 5 was 0.16 μJ at 18.6 mm of deformation, 62 times smaller than the cylinder having 9.9 μJ at 10.68 mm of deformation.

In summary, as the number of convolutions increases, the axial stiffness decreases, while the contraction ratio increases. Finally, among the evaluated structures, Type 3 (having four convolutions) was identified as the optimal one. Indeed, it showed not only relatively larger contraction ratio with respect to Type 1, 2 and simple cylinder, but also a much higher strain energy than Type 4 and 5, as the graphs of Fig 3 demonstrate.

#### 2.2.2 Reinforcement of axial stiffness

Indeed, if using only the optimized elastomeric skin, the bellow axial stiffness is still quite weak with respect to maximum axial load, because the skin itself cannot support its own weight due to the square-cube law. Namely, once the skin is scaled up, the volume increases with the cubic power, while the cross section increases with the square value only.

Soft PAMs using elastomeric materials cannot be scaled up without providing additional axial stiffness enhancement. However, the utilization of composite materials or structures (*i*.*e*., extensible or inextensible sheath or fabric [40,41], granular material [42], liquid metal [43], a constraint frame structure [44], etc.) increases the weight/mass of actuator (as well as of its fabrication complexity), resulting in lower energy density or limited deformation. In particular, in the case of a PAM operated by negative pressure, the stiffer the structure, the higher the pressure required to achieve a desired deformation. For the same reasons, the use of compression springs, usually made of very stiff materials (*i*.*e*., metals, etc.), inside the structure is not suitable.

In contrast to such high stiff materials, the open-cell foam, consisting of ensnared bubbles, is highly deformable and ultralight. Moreover, it can be employed in PAMs designs by encasing them with an elastomeric skin. In our previous studies [37,45], the viscoelastic behavior of open-cell foam was investigated, and three phases were observed: a) linear elastic region; b) plateau region; and c) densification region. In the latter regime, the axial stiffness increased significantly because the constituent microfibers are packed due to an external load. Then, the actuator does not contract relevantly when the foam reaches the densification region (after 40% strain). Based on such foam characteristics, the aim was to design a bellow-type PAM able to contract up to 50%.

Nevertheless, since the negative pressure causes volume shrinking, the bellow deforms in both radial and longitudinal directions. This limits the linearity of the relation between the deformations and the applied pressures. To reduce this effect, Plexiglas^®^ rings with a thickness of 0.8 mm were added at each convolution. As it will be detailed later, these rings affect only the 10% of the total weight; moreover, an additional stiffer skin material (Dragon Skin^TM^ 30) was employed to further investigate the best actuator performance.

### 2.3 Structural analysis

The critical load to cause the buckling of the skin needed to be evaluated in order to ensure the reliable and accurate operation of the proposed PAM. In the following sections, the buckling analysis and a quasi-static model are presented to approximate the blocking force versus the negative pressure.

#### 2.3.1 Buckling analysis

The bending stiffness of the bellow (*C*_*bg*_) is proportional to axial stiffness [46–48], which can be derived as

$$C_{bg}=EI=\frac{(f_{iu})∙D_{m}^{2}∙h}{8}$$ (1)

where *E* and *I* are the Young’s modulus of the material and the moment of inertia of the structure, respectively; *f*_*iu*_ refers to the axial stiffness, and *D*_*m*_ and *h* are the mean diameter and pitch, respectively. Even if Eq (1) is rigorously correct only for isotropic materials, here it is used to approximate also the behavior of hybrid structures, by considering an equivalent mean Young’s modulus fitted from experiments. Then, the buckling pressure (*P*_*c*_) can be described by:

$$P_{c}=\frac{\pi ∙C_{bg}}{D_{m}^{2}{∙h}^{2}}$$ (2)

In order to derive the axial stiffness (*f*_*iu*_) through a cyclic compression test, a linear numerical fitting using the Least Square Method (LSM) was performed, and the slope of each fitting was exploited. Here, we assumed that the relation between the strain and the average slope corresponds to the axial stiffness of the viscoelastic structure. Finally, the parameters that were investigated to compare the PAM performance are summarized in Table 3.

**Table 3:** Mechanical parameters and results of the buckling analysis.

Nomenclature	Dimension	Case 10	Case 10/rings	Case 30	Case 30/rings
Axial stiffness (*f*_*iu*_)	[N/mm]	0.103	0.14379	0.1673	0.20779
Bending stiffness (*C*_*bg*_)	[N∙mm^2^]	245.632	342.836	398.974	495.5337
Theoretical buckling pressure (*P*_*c*,*theo*_)	[kPa]	2.3793	3.321	3.8645	4.80
Experimental buckling pressure (*P*_*c*,*exp*_)	[kPa]	2.482	3.309	3.760	4.891
Error rate (*e*)	[%]	1.63	0.367	2.788	1.859

#### 2.3.2 Quasi-static analysis of the blocking force

The blocking force of the actuator can be approximated by using a quasi-static analytical model [27,34]. To simplify the analysis, in Eq (3) the assumption is that the work produced by the distributed pressure acting on the inner wall of the skin equals the work required to lift a weight:

−F∙dh=P∙dv (3)

where *F* and *dh* are the blocking force owing to the applied negative pressure and the linear displacement, respectively. *P* and *dv* are the gauge pressure and the change in the volume of the actuator, respectively. Similar to Eq (2), also Eq (3) is used for all investigated structures, where the friction between the foam and the skin, the foam density (much lower than the skin one), and the viscosity of working fluid (air) are neglected. To derive the function of compressive force for the displacement, the arc-length of an ellipse (*l*) is approximated with a circular length (*l*_*0*_ = πr). Then, the length of the major axis (α) can be approximated as:

$$\alpha ≅\sqrt{\left(\frac{2l_{0}^{2}}{\pi^{2}}-\frac{h^{2}}{4}\right)}$$ (4)

Moreover, the differential with respect to *h* can be written in Eq (5), which indicates the ratio of the contraction in radial and longitudinal directions (*C*_*R*_):

$$C_{R}=\frac{d\alpha }{dh}=-\frac{1}{4}h\frac{1}{\sqrt{\left(\frac{2l_{0}^{2}}{\pi^{2}}-\frac{h^{2}}{4}\right)}}=-\frac{h}{4\alpha }$$ (5)

Therefore, the function of displacement and volume for the blocking force can be written by the following partial differentiation:

$$\frac{dv}{dh}=\frac{\pi D^{2}}{8}-\frac{2\pi }{3}\alpha h∙\frac{d\alpha }{dh}-\frac{\pi }{3}\alpha^{2}$$ (6)

Finally, the blocking force as a function of displacement (*h*) can be derived by exploiting Eqs (5) and (6), as:

$$F(h)=-P∙\frac{dv}{dh}=-P∙\left[\frac{\pi D^{2}}{8}+\frac{\pi h^{2}}{6}-\frac{\pi }{3}\alpha^{2}\right]$$ (7)

### 2.4 Fabrication

**Fig 4:**
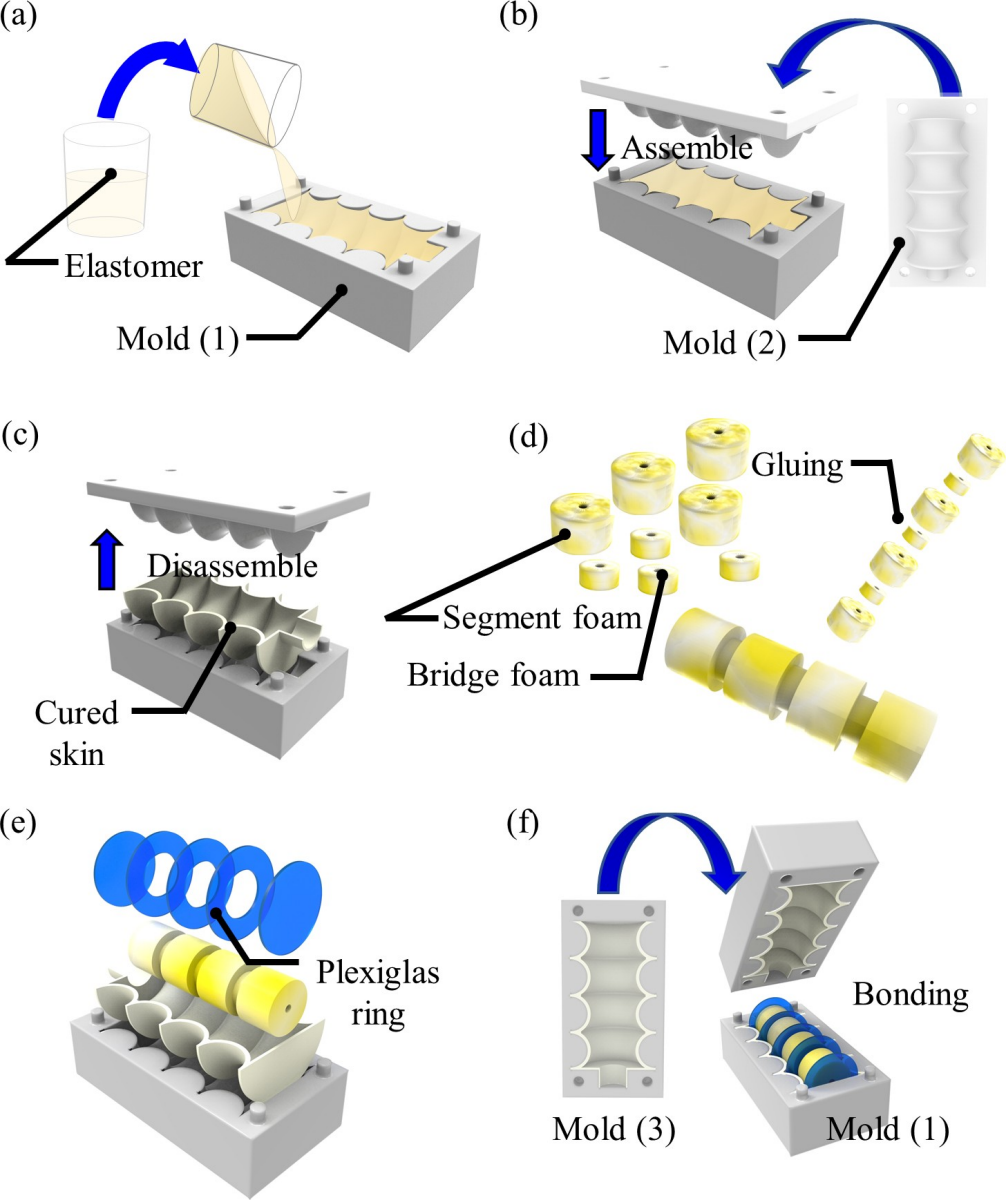
Fabrication procedure: For the two halves of the skin (a) pouring of specific elastomer in 3D printed mold (1); (b) mold cover (2) assembling; (c) after curing, the skin is detached from the molds; (d) foam elements are bonded using a silicone epoxy; (e) foam segments and Plexiglas^©^ rings are integrated into one-half skin by a mold; (f) mold (3) is used to host the second half skin and for bonding with the assembled structure.

The fabrication process of the UH-PAM is depicted in Fig 4. A commercial polyurethane open-cell foam (8643K549, McMaster-CARR, Chicago, USA) sheet was used to cut different cylindrical segments (four having 20 mm diameter and 12.4 mm length, and three having 11 mm diameter and 6.2 mm length). Five Plexiglas^®^ rings (having a diameter of 28.5 mm and thickness of 0.8 mm) were patterned by 2D laser cutter (Versa LASER VLS 3.5). Dragon Skin^TM^ 10 or 30 part A and B were manually mixed with a weight ratio of 1:1 and degassed in a vacuum chamber. Then, the mixed liquid was poured into molds made by 3D printing (ProJet MJP 3600, 3D Systems, South Carolina, US), in order to cast the two halves of the overall elastomeric skin. After curing for 15 hours, the skins were detached from the molds, as shown in Fig 4C. The foam structure was built by bonding all the elements with silicone adhesive (SIL-Poxy® adhesive, Smooth-On Inc., Macungie, PA, USA), as shown in Fig 4D. Since the foam absorbs silicone, all the skin parts were bonded to the foam elements by brushing the same silicone material of the skin. After curing of the silicone, both halves of the skin were bonded together, as shown in Fig 4F. Additional details for fabrication are available at our laboratory protocol (dx.doi.org/10.17504/protocols.io.bi8bkhsn).

### 2.5 Experimental setups

**Fig 5:**
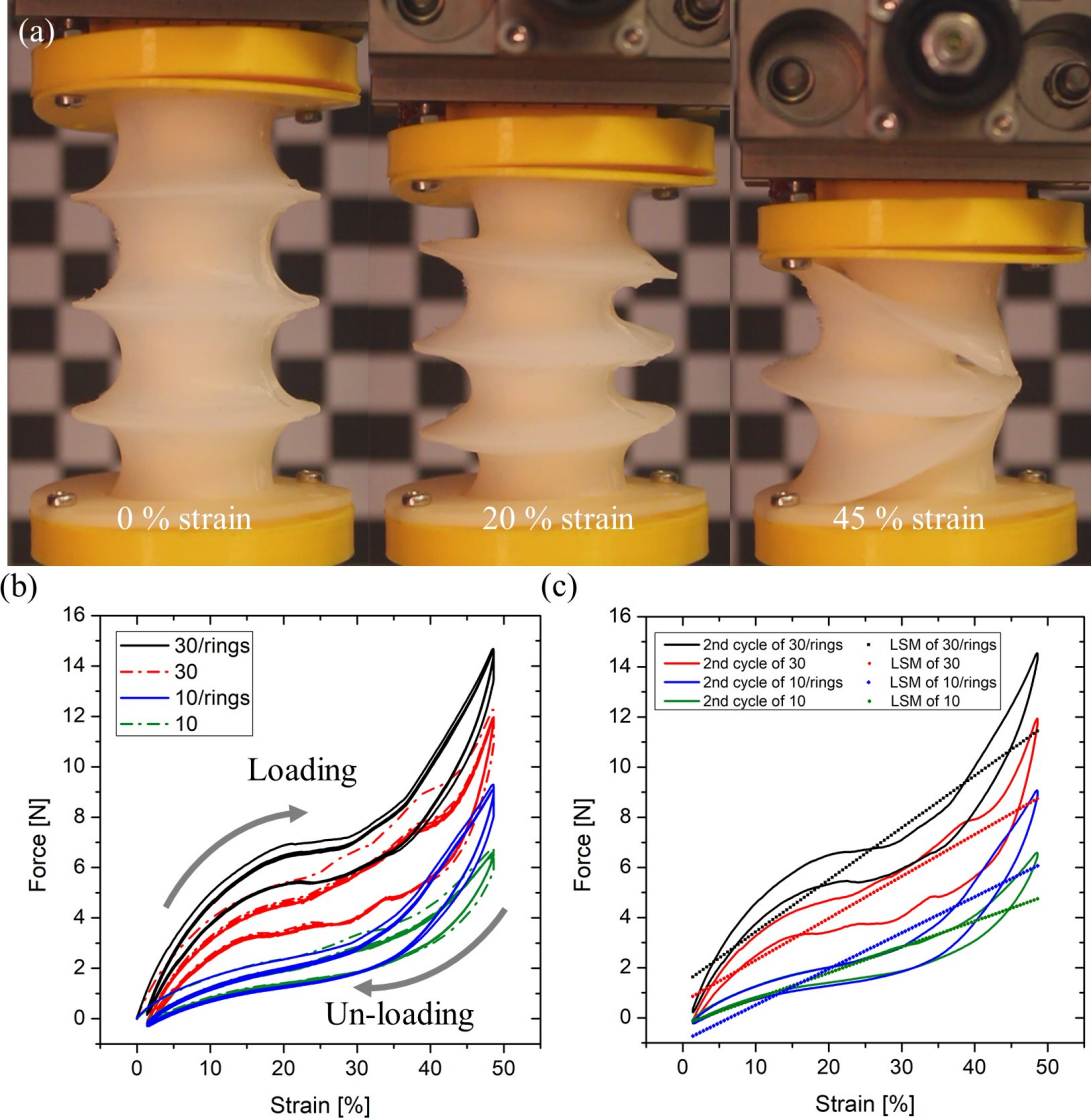
Cyclic compression test results: (a) images showing the compression testing at different strain phases; (b) the experimental results of loading/unloading cycles for each case; (c) linearly interpolated lines (LSM) for each case.

The cyclic compression tests of different PAM designs were performed using a universal material testing machine (Z005, Zwick/Roell, Ulm, Germany), as shown in Fig 5A, and loading-unloading curves were obtained for each PAM case by running ten cycles. In this experiment, the pre-load setting was 0.01 N, and the velocity was 200 mm/min, with a maximum displacement of 35 mm.

As described in the next sections, the optimal PAM structure selected after the cyclic compression testing was further characterized for its static and dynamic behavior by the following experimental setup. The vacuum pressure for the actuation was controlled by a vacuum flow regulator (ITV0090, SMC Corp., Tokyo, Japan) having a 0.5 s response time. Furthermore, the vacuum pump (Diaphragm Vacuum Pumps and compressor, N022 AN.18, KNF LAB, Balterswil, Switzerland) could generate a vacuum up to 100 mbar. A LabVIEW^®^ program was developed for data acquisition and to generate the analog signal through a DAQ board (USB 6218, National Instruments, TX, USA). The kinematics of the actuator was monitored by 6 DoFs tracking system with an electromagnetic localization probe (AURORA^®^ EM, Northern Digital Inc., Waterloo, Canada). To investigate the blocking force, both terminals of the soft actuators were clamped by rigid mechanical parts that were clamped. Finally, the force was monitored by a load cell (3001493 Xforce Load cell, F max 50 N, Zwick/Roell, Ulm, Germany), with a negative pressure applied from 0 kPa to -80 kPa, with steps of -5 kPa.

## 3. Experimental results

### 3.1 Cyclic compression tests

Cyclic compression tests were performed in order to investigate the buckling pressure causing lateral squirm effects, as well as to analyze the mechanical behaviors of the actuator prototypes with respect to an externally applied load. The experimental buckling pressure was compared to the theoretical values. Different structures were investigated experimentally, as detailed in Table 3, namely: cases 10, 30, 10/rings and 30/rings, were fabricated with Dragon Skin^TM^ 10, 30, and with added rings, respectively. As derived from Eqs (1) and (2), the axial stiffness of the specific structure depends on both the bellow geometry and the Young’s modulus of the skin material.

The resulting characteristics (*i*.*e*., axial stiffness and hysteresis) of each structure are shown in Fig 5B. Among them, case 30/rings shows the minimum hysteresis and the maximum axial stiffness. In general, the axial stiffness increases with the stiffness of the skin material (*i*.*e*., cases 30 and 30/rings compared to case 10 and 10/rings), and the presence of the rings (cases 10/rings and 30/rings) shows an increased axial stiffness while a decreased hysteresis, as plotted graph of Fig 5C. In summary, the axial stiffness of case 30/rings is around 0.208 N/mm, having 4.9 kPa of buckling pressure. Accordingly, the bellow-type case 30/rings, namely Ultralight Hybrid Pneumatic Artificial Muscle (UH-PAM), can support up to around 6.8 N load axially, which is 34.5 times greater than its own weight (20 g). In particular, the UH-PAM shows more uniform deformation than other cases when a high negative pressure drives it to full contraction.

### 3.2 UH-PAM static characterization

**Fig 6:**
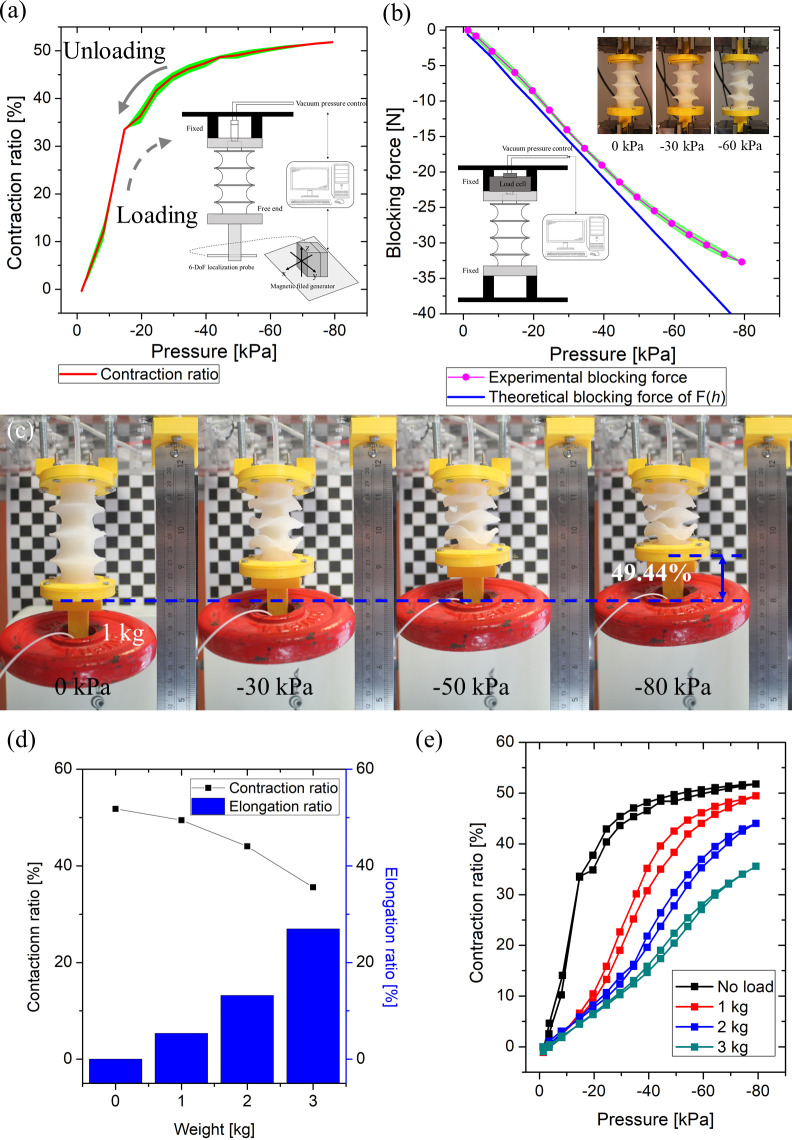
Experimental results of static and quasi-static characteristics of the UH-PAM: (a) contraction ratio versus pressure characteristics; (b) comparison between the experimental (pink) and theoretical (blue) blocking force; (c) sequence of images showing lifting of 1kg lifting by negative pressure; (d) comparison of contraction and elongation ratio according to the attached weight from 1 to 3 kg; (e) contraction ratio versus pressure graph according to the attached weight from 1 to 3 kg.

The static behavior of the proposed UH-PAM was characterized and compared with the quasi-static numerical model. The characterization results are shown in Fig 6A and 6B. The blocking force is linearly proportional to the applied negative pressure, with a maximum blocking force of 32.7 N at -80 kPa. As shown in Fig 6B, there is a good agreement between experimental data (black squared marks) and the quasi-static model (blue line), with an error less than 10.35% up to -65 kPa, and a maximum error of 27.38% at -80 kPa. These results verify the assumption of “ellipse constant arc-length” in the quasi-static model (section 2.3.2). To further investigate the actuation performance, a weight was attached at the free end of the UH-PAM, ranging from 1 to 3 kg. The proposed UH-PAM is capable of 49.4% of contraction ratio at -80 kPa, when lifting 1 kg weight. It was observed that the UH-PAM can lift a weight up to 3 kg without tearing of the skin (Fig 6E), while the contraction ratio is decreased to 32.3%.

Finally, Table 4 lists the UH-PAM specifications, the portion of each component’s weight (*i*.*e*., rigid rings, foam, and the skin), the energy density (*i*.*e*., blocking force ×deformation versus total weight), and the strength density (*i*.*e*., blocking forces versus total weight), and other parameters.

**Table 4:** Specifications of UH-PAM.

	Dimensions [portion, %]
Total weight [g]	20 [100]
Rings [g]	2 [10]
Foam [g]	1.3 [6.5]
Skins [g]	16.7 [83.5]
Max. deformation [mm]	40.4 [51.79]
Energy density [W/kg]	66.3
Strength density [kN/kg]	1.64

### 3.3 UH-PAM dynamic characterization

**Fig 7:**
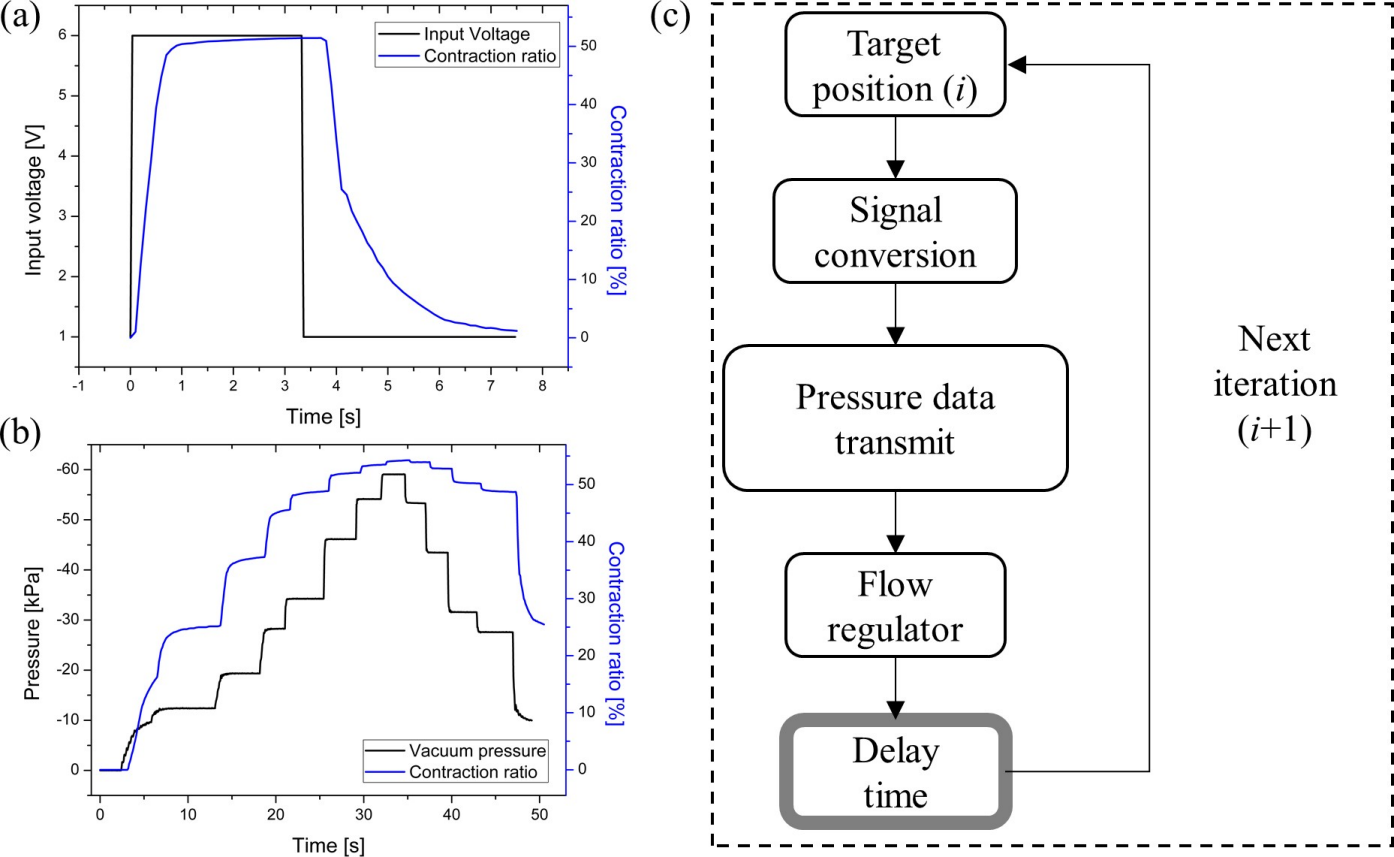
Dynamic characterization results: (a) step response from 0 to 6 V; (b) stepwise response with random time spans for the pressure versus the contraction ratio; (c) block diagram for open-loop control of the UH-PAM.

The dynamic characteristics of the UH-PAM were evaluated by varying the input voltage of the flow regulator with a square wave from 0 to 6V, as shown in Fig 7A, and by measuring both displacement and pressure. The actuator was not electrically driven, however in our setup the control parameter was the input voltage of the flow regulator, which was proportional to the input pressure. The results indicated how the deformation ratio had a different behavior for the contraction and the release phase, within a full span, as shown in Fig 7A and 7B (stepwise response with random time spans), while no overshooting or undershooting was presented. Indeed, the UH-PAM contraction time (i.e., time to reach 90% of full contraction, measured from the curve shown in Fig 7A) was 0.5 s. On the other hand, the time to recover from a full contraction to the initial position was of 3.8 s (for the full contraction/release behavior, see S1 Video). Both delays were mainly due to the response time of the flow regulator that limited the maximum flow rate to set the inner pressure.

In the contraction phase, the response was faster since the input of the flow regulator had a negative pressure deriving from the vacuum pump. Oppositely, in the release phase, the UH-PAM internal volume was expanded only by atmospheric pressure (then with a lower pressure difference with respect to the previous case), causing a longer delay. In principle, to make the release phase faster, a positive pressure could be applied to the UH-PAM, at the cost of increasing the complexity of the control system.

This behavior led to a relevant hysteresis (up to 100%), typical of such kind of soft actuators. To address this issue, the actuator motion could be limited to a shorter contraction range, with the result of a relevantly reduced delay. For instance, below -10 kPa, corresponding to a contraction of ~ 10%, the delays for both contraction and release phases are less than 1 s. For this reason, single steps of 0.1 kPa in both phases were applied, with a delay time of 500 ms for each command, as illustrated in the flow diagram of Fig 7C (similar to the approach addressed in [49]). In addition to this, the dynamic frequency for UH-PAM contraction/release can be appropriately tuned to reduce the hysteresis, allowing a more accurate control. Finally, we used the UH-PAM to actuate a 1 DoF joint (see Figure A and B in S1 Text), and to investigate the dynamic responses for triangular input functions having different frequencies. For this platform, both actuation range and frequency were tuned to minimize hysteresis and to obtain precise movement. As detailed in SI, hysteresis can be reduced to 11.5%, and the desired deformations at fully contracted and released states were successfully achieved.

### 3.4 UH-PAM based Stewart platform

**Fig 8:**
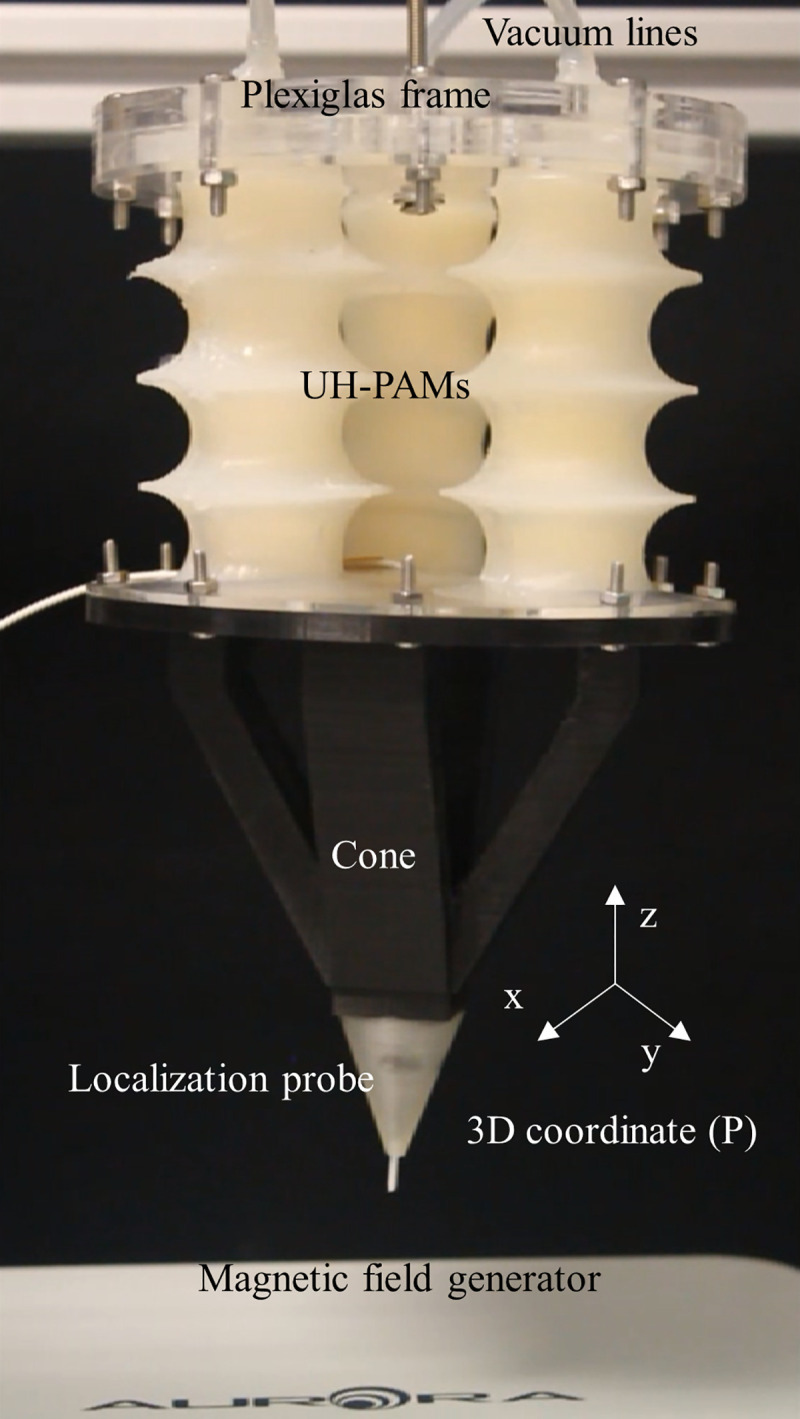
Configuration of the 3DoFs Stewart platform integrating n.3 UH-PAMs, a fixed joint, a rigid cone, and the AURORA^®^ tracking system with the 3D coordinate system.

**Fig 9:**
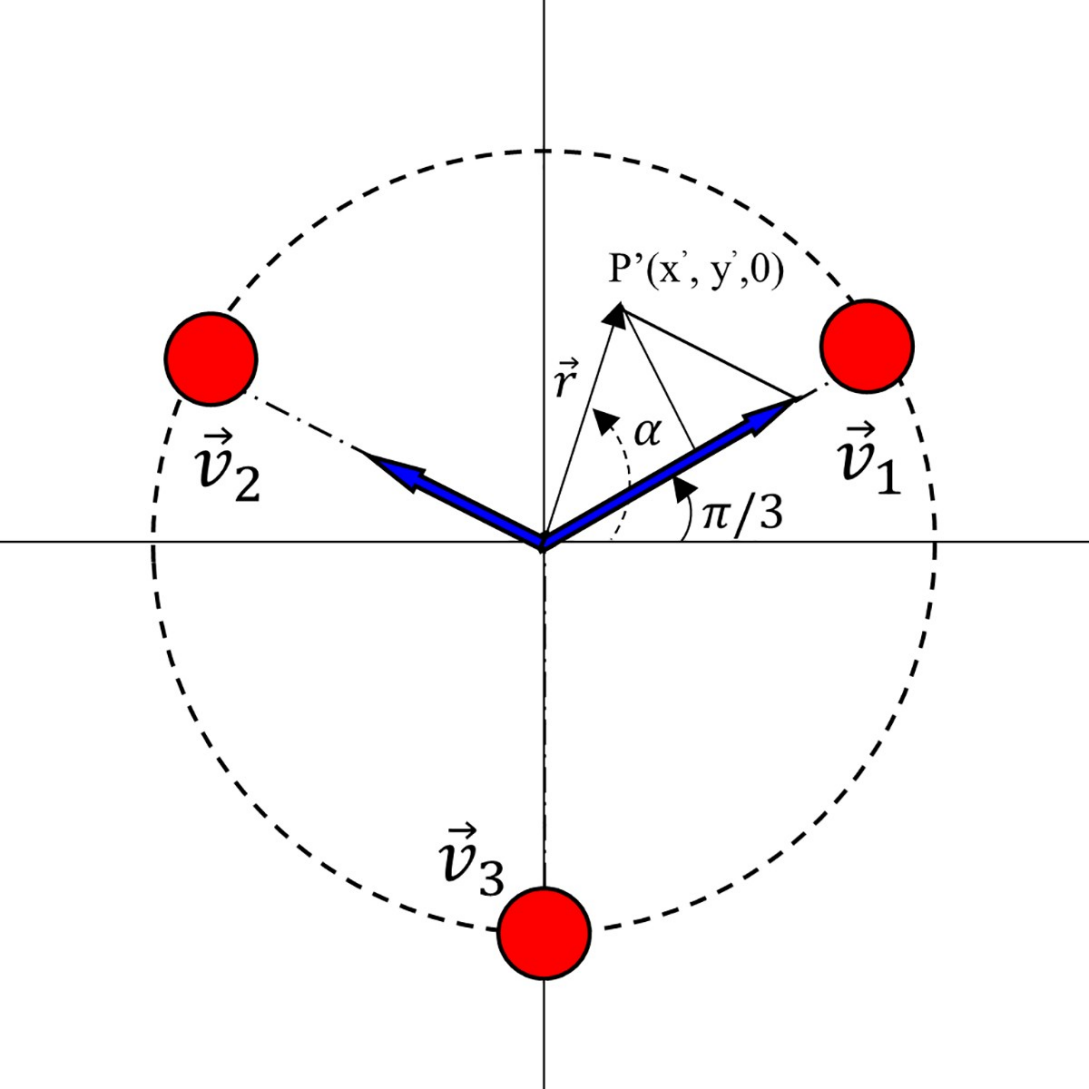
Scheme of the 3D coordinate of the projected vector *r* for the orientation of the Stewart platform.

A Stewart platform with 3 DoFs was designed and built with open-loop control. The platform (shown in Fig 8) consisted of three UH-PAMs arranged at 120° from each other, as depicted in Fig 9.

Each actuator was connected to a vacuum line and supported by a Plexiglas^©^ frame, at the top side. At the bottom side, they were attached to a plastic cone, which at its tip embedded the localization probe of the tracking system. Accordingly, by varying the vacuum in each UH-PAM, different displacements or orientations of the bottom cone can be smoothly generated. The 3D coordinates of the cone tip were retrieved by the tracking system, and a geometry mapping algorithm based on an open-loop system was programmed. To simplify the control, two assumptions were made: 1) the relation between the displacement and pressure for a full contraction/release span is linear; 2) the hysteresis is negligible, meaning a one-to-one function between the pressure and the desired displacement.

As already shown in the static and dynamic characterizations of a single UH-PAM, the error can be reduced relevantly by controlling the system with single steps of -0.1 kPa and a delay of 0.5 s for each command.

For this platform, a negative pressure range between -10 kPa and -60 kPa was used to perform the movement. The 3D coordinates (P) of the cone tip were projected into the *x-y* plane, obtaining simplified 2D coordinates (P’). As a result, a single command generated a movement operated by the simultaneous actuation of only one or two UH-PAMs. For example, if the projected coordinate P’ is placed in the first quadrant, as shown in Fig 9, the vector *r* can be derived by the combination of *v*_*1*_ (*i*.*e*., actuation of UH-PAM 1) and *v*_*2*_ (*i*.*e*., actuation of UH-PAM 2) vectors, with *v*_*3*_ set to zero (*i*.*e*., UH-PAM 3 not activated).

The magnitude of the vector *r* can be written as:

$$\left[\begin{array}{c} \left|\vec{v_{1}}\right|\\ \left|\vec{v_{2}}\right|\\ \left|\vec{v_{3}}\right| \end{array}\right]=\left[\begin{array}{ccc} \cos\left(\alpha -\frac{\pi }{3}\right)+\frac{1}{\sqrt{3}}sin(\alpha -\frac{\pi }{3})*cos(\alpha -\frac{\pi }{3}) & 0 & 0\\ 0 & \frac{2}{\sqrt{3}}sin(\alpha -\frac{\pi }{3})*cos(\alpha -\frac{\pi }{3}) & 0\\ 0 & 0 & 0 \end{array}\right]∙\left[\begin{array}{c} \left|\vec{r}\right|\\ \left|\vec{r}\right|\\ 0 \end{array}\right]$$ (8)

**Fig 10:**
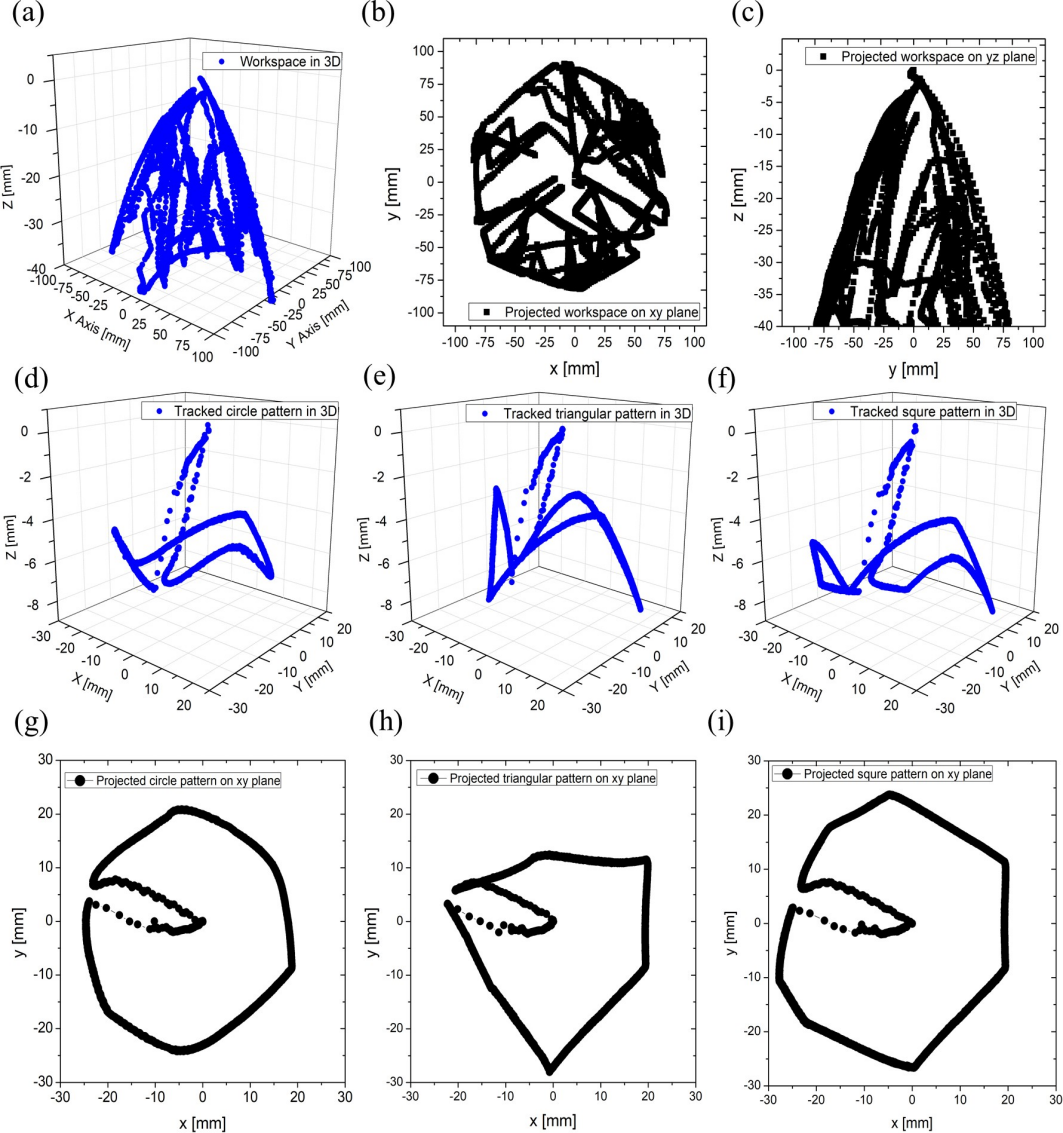
Results of 3DoFs Stewart platform performance: (a) workspace by joy stick in 3D; (c) workspace in yz plane; (c) workspace in xy plane; (d) tracked travel of circle drawing in 3D; (e) tracked travel of triangle drawing in 3D; (f) tracked travel of rectangle drawing in 3D; (g) tracked travel of circle drawing in 2D; (h) tracked travel of triangle drawing in 2D; (i) tracked travel of rectangle drawing in 2D.

The control system was implemented in LabVIEW^®^. During the calibration phase, the full 3D workspace was explored by moving the tip with a joystick (PlayStation navigation controller, Sony, Tokyo, Japan), as shown Fig 10A to 10C. Also, this system was interfaced to a maze platform (hosted on top of the Stewart platform in the upside-down position) to test the manual control (see S2 Video). Then, different handwriting figures in the 2D plane (*i*.*e*., circle, triangle, and square) were pursued by utilizing the open-loop-based geometry algorithm, as shown in Fig 10G to 10I (see also S3 Video).

In this case, a trajectory error up to 20.9% is observed (see Figure A in S2 Text for further details). Also, by using the system without the cone and tracking system embedded, a heart-like trajectory was performed as an example of more complex geometry (see S4 Video).

## 4. Discussion and conclusion

Typical constituent materials of conventional bellow-type PAMs are metallic (*i*.*e*., stainless, aluminum, etc.) with Young’s moduli in the GPa range. They are being widely used in industrial applications [50,51] due to their high contraction and/or elongation ratio. Indeed, bellow structures offer an opportunity to develop also PAMs based on soft materials, showing several promising characteristics (*i*.*e*., versatility, compliance, etc.). However, these cannot be directly reflected in structures built with soft and/or thin-wall structure since buckling failure can occur with quite low payloads, owing to the lack of axial stiffness.

In this work, a vacuum-powered PAM, having a bellow configuration with 51% of contraction ratio, is introduced. In particular, an innovative solution is proposed for building a structural backbone, *i*.*e*., soft open-cell foam segments are merged with thin Plexiglas^©^ rings (both contributing to 16.5% of the total weight) inside a soft skin.

Adding a ring at each UH-PAM convolution (case 30/rings) causes the increase of both, the buckling load and the axial stiffness, to up to 6.7 N and to 24.2%, respectively, if compared with the same structure without rings (case 30). The axial stiffness was further increased up to 62% by using Dragon Skin^TM^ 30 (case 30), instead of Dragon Skin^TM^ 10 (case 10). The axial stiffness and the buckling pressure of the proposed UH-PAM result in 0.208 N/mm, and 4.9 kPa, respectively. Therefore, a good trade-off between the needed mechanical behavior and the overall structure compliance is found.

An experimental platform to investigate the static characteristics of UH-PAM was developed, and a quasi-static model was derived to approximate the blocking force. As a result, the UH-PAM, having a weight of 20 g, can lift a payload up to 3 kg, which is very close to the value of 3.3 kg, derived from the quasi-static model. Furthermore, the total weight of the proposed UH-PAM is lower than both OV-PAM (53 g) [27] and single module of SPA (28 g) [52]. Regarding the constituent materials, foam and Plexiglas^©^ rings represented only 6.5% and 10% of the total weight, respectively. The energy density and the strength density of the UH-PAM were 66.3 W/kg and 1.64 kN/kg, respectively. These values are very competitive with previous works (*i*.*e*., McKibben [22], rPAMs [23], LSOVA [34]), and comparable to most of FOAMs having the strength density up to 2 kN/kg. In particular, the UH-PAMs can be actuated by using relatively low vacuum pressure (up to -80 kPa), which implies a good energy efficiency. UH-PAM requires very small pressure to generate significant deformation. Indeed, as shown in the graph of Fig 6E the UH-PAM showed most of its contraction and elongation ratio (~40%) in the lower pressure range (under -20 kPa), with a very linear behavior. However, after this transition value, the characteristics becomes non-linear. In addition, this pressure transition value can vary with the applied payload (in particular in the 1–3 kg range), as similarly observed in most of the surveyed PAMs [23,27,34]. This means that precise control for the whole contraction range with unknown payloads is limited in an open-loop system. This issue could be overcome by providing sensory feedback on the UH-PAM movement, in order to implement closed-loop control.

The dynamic characterization resulted in a quite large hysteresis. Such viscoelastic behavior is due to the foam used in these prototypes. However, as already demonstrated in our previous works [53,54], foam mechanical properties can be finely tuned (*i*.*e*., varying the porosity and/or constituent material) with custom-made structures, obtaining porous materials with proper softness and good linearity.

Dynamic characterization also showed different response times for the two phases of contraction (0.5s) and release (3.8 s). The worse performance in the release phase is mainly due the flow regulator using only atmospheric pressure. In principle, this time can be reduced relevantly by applying a positive pressure, although it would increase the system complexity. In order to investigate cyclic motion with an optimal frequency, an arm platform with 1 DoF was developed (see SI), and smooth motion without a stroke loss was obtained at 1/55 Hz. Also, in this case, positive pressure in the release phase would improve the system performance.

Furthermore, a 3 DoFs Stewart platform with a simplified geometry algorithm was investigated. In this case, a proper delay time for each command was introduced to implement an open-loop control, and different handwriting figures were considered, obtaining an average trajectory error up to 20.9%. This error is quite acceptable in the soft actuation field, and it is mainly due to the limits in the assumptions done in the control algorithms (*i*.*e*., linearity in the full span range and negligible hysteresis). As already discussed above, the precision could be improved by embedding proprioceptive sensing capabilities. This would enable investigating sensing-actuation-control trade-offs needed to address different applications like soft manipulation or soft wearable assistive devices.

In the near future, in addition to integrating soft sensors for proprioception, the proposed concept will be further improved by developing a graded structure with different soft materials. Finally, the UH-PAMs could provide insights to develop soft robotic systems for high energy-efficient and high lifting payload-oriented applications, such as assistive robots, surgical equipment, or wearable exoskeletons.

## Supporting information

S1 Table Parameters of the skin Mooney-Rivlin hyperelastic model. (PDF)

S1 Text 1 DoF arm platform. (PDF)

S2 Text Error estimation in 3 DoFs Stewart platform. (PDF)

S1 Video The dynamic behaviour of UH-PAM is shown by applying a step input voltage. (WMV)

S2 Video The 3DoFs Stewart platform is tested with manual control by means of a joystick. The details of the system are provided in Section 3.4 of the manuscript. For demonstration purposes a maze is housed on top of the platform.
(WMV)

S3 Video The 3DoFs Stewart platform is demonstrated with open-loop control. The vacuum in each UH-PAM is smoothly controlled in order to draw a circle in the XY plane. The details of the system are provided in Section 3.4 of the manuscript.
(WMV)

S4 Video The 3DoFs Stewart platform is demonstrated with open-loop control. A more complex figure (heart-like) is shown. The details of the system are provided in Section 3.4 of the manuscript.
(WMV)
