# Supplementary material for: Development of the Ultralight Hybrid Pneumatic Artificial Muscle: Modelling and optimization
Source: PLoS One. 2021 Apr 22;16(4):e0250325. doi: 10.1371/journal.pone.0250325 (PMC8062031; doi:10.1371/journal.pone.0250325)
Supplement: S1 Table — (PDF) [file pone.0250325.s001.pdf]

S1 Table. Parameters of the skin Mooney-Rivlin hyperelastic model.

Parameters	Dimensions [Pa]
C10	$-2.1242 \cdot 10^{5}$
C01	$2.2114 \cdot 10^{5}$
C20	$1.1528 \cdot 10^{7}$
C11	$-2.4705 \cdot 10^{7}$
C02	$1.3631 \cdot 10^{7}$
C30	72.253
C21	-1583
C12	$-2.8688 \cdot 10^{6}$
C03	$1.8054 \cdot 10^{6}$
