# Supplementary material for: Development of the Ultralight Hybrid Pneumatic Artificial Muscle: Modelling and optimization
Source: PLoS One. 2021 Apr 22;16(4):e0250325. doi: 10.1371/journal.pone.0250325 (PMC8062031; doi:10.1371/journal.pone.0250325)

S1 Text

1 DoF arm platform

A 1 DoF platform was implemented integrating one UH-PAM, as shown in Fig A in S1 Text. The goal was to achieve a smooth motion without a stroke loss, by proper UH-PAM control. A lever arm was built by using a 8 mm thick Plexiglas® and a passive polystyrene hand was attached at its end. The applied vacuum to the UH-PAM was from -10 kPa to -60 kPa, and the joint motion was tracked by means of an AURORA® system attaching the probe at the tip of the hand. Triangular functions for varying inlet pressure with different frequencies (from 0.013 to 0.1 Hz) were applied, as shown in Fig Ab to f. For each frequency, after the first cycle, A and B mark the release and fully contraction points, respectively. Then the range of motion was evaluated for each case. For higher frequencies, a stroke loss in the movement was observed.

Indeed, as shown in Fig. Aa, in case of 0.1 Hz (red line) the A point indicates a lower motion range with respect to 0.018 (1/55) Hz (black line). In addition, the same range of motion was observed at 0.013 Hz.

Hence, 1/55 Hz is the optimal control frequency for this platform. In this case, the hysteresis can be reduced to 11.5%, as shown in Fig B in S1 Text. We can conclude that by increasing the actuation frequency, a greater stroke loss can be observed because the releasing of UH-PAM requires a longer time, and this sets a limit on the actuation velocity for proper control movement.

Figure A. 1 DoF arm platform demonstration: (a) In the inset, a photograph of the arm platform performing. Points A (releasing position) and B (full contraction position) are for actuation frequency of 1/55 Hz (black line) and 1/10 Hz (red line), respectively.

Pressure cycles with triangular function of 1/10 Hz (b), 1/18 Hz (c), 1/38 Hz (d), 1/55 Hz (e), and 1/75 Hz (f).

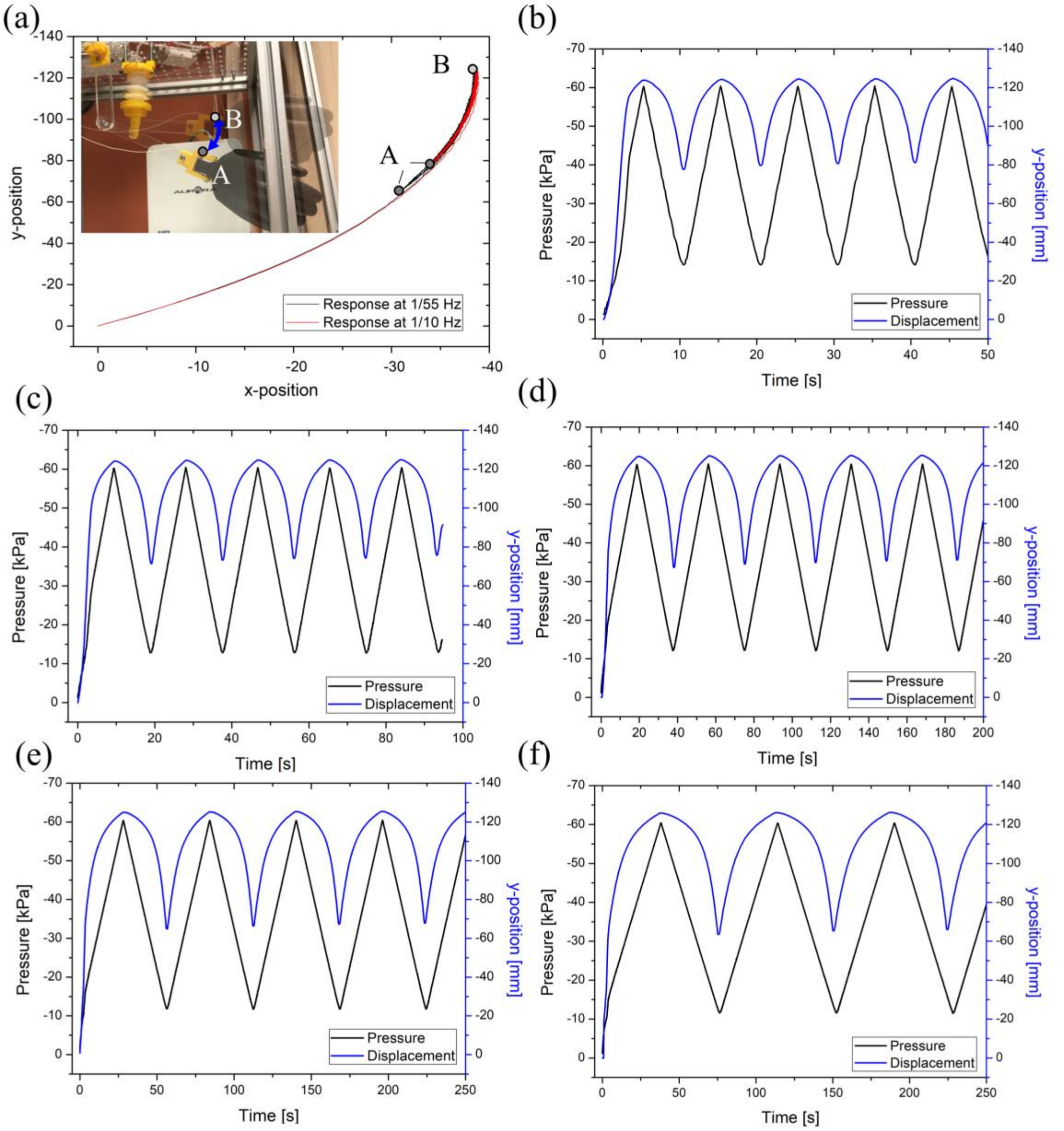

Figure B. Tracked y-position at 2nd cycle for 1/55 Hz applied frequency, showing a hysteresis reduced to 11.5%.

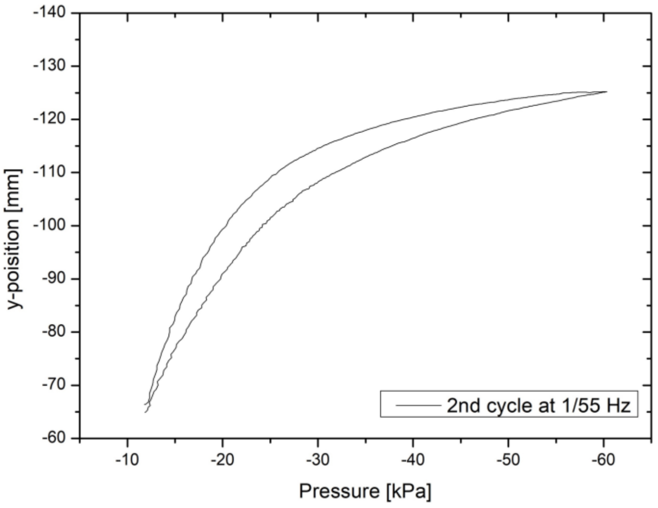

Supplement: S1 Text — (PDF) [file pone.0250325.s002.pdf]
