# Supplementary material for: Development of the Ultralight Hybrid Pneumatic Artificial Muscle: Modelling and optimization
Source: PLoS One. 2021 Apr 22;16(4):e0250325. doi: 10.1371/journal.pone.0250325 (PMC8062031; doi:10.1371/journal.pone.0250325)

S2 Text

Error estimation in 3 DoFs Stewart platform

In order to estimate the open-loop control precision, a trajectory error analysis was performed for each handwriting scheme. The relative error for each figure was calculated by comparing the real area drawn by tracked data with its corresponding reference target area. In this way, an average value of 11.8%, 20.9%, and 17.4% was retrieved for the circle, the triangle, and the square, respectively. In Fig A in S2 Text, a sketch comparing the real pattern (red dots) with the targeted trajectory (black solid lines) is shown for each figure.

Figure A. **Comparison between the real pattern (red dots) obtained with the Stewart platform and the targeted trajectory (black lines)** for (a) circle, (b) triangle, and (c) square.

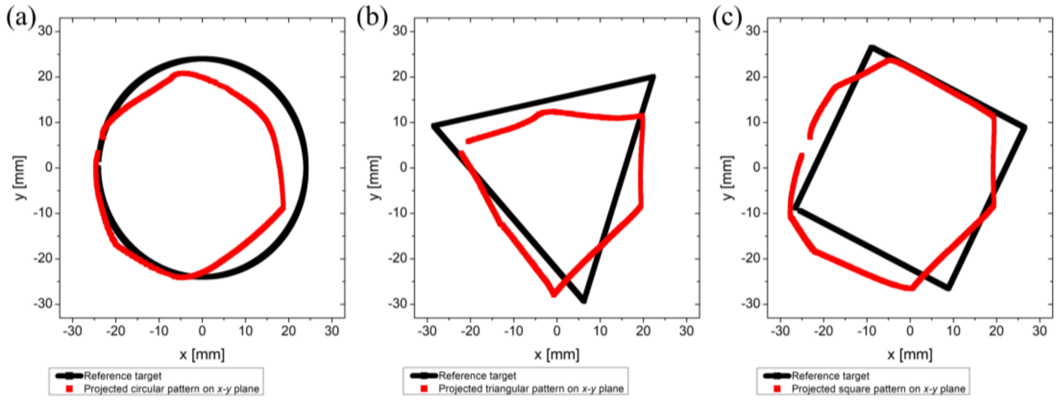

Supplement: S2 Text — (PDF) [file pone.0250325.s003.pdf]
